# Improved methanol tolerance of *Rhizomucor miehei* lipase based on N‑glycosylation within the α-helix region and its application in biodiesel production

**DOI:** 10.1186/s13068-021-02087-6

**Published:** 2021-12-15

**Authors:** Miao Tian, Lingmei Yang, Zhiyuan Wang, Pengmei Lv, Junying Fu, Changlin Miao, Ming Li, Tao Liu, Wen Luo

**Affiliations:** 1 Key Laboratory of Renewable Energy, Guangzhou Institute of Energy Conversion, Chinese Academy of Sciences, Guangzhou, 510640 People’s Republic of China; 2 University of Chinese Academy of Sciences, Beijing, 100049 People’s Republic of China; 3 Guangdong Provincial Key Laboratory of New and Renewable Energy Research and Development, Guangzhou, People’s Republic of China; 4 Guangdong Provincial Key Laboratory of Pharmaceutical Bioactive Substances, Guangdong Pharmaceutical University, Guangzhou, 510006 People’s Republic of China

**Keywords:** *Rhizomucor miehei* lipase, α-Helix region, N‑Glycosylation, Methanol tolerance, Biodiesel

## Abstract

**Background:**

Liquid lipases are widely used to convert oil into biodiesel. Methanol-resistant lipases with high catalytic activity are the first choice for practical production. *Rhizomucor miehei* lipase (RML) is a single-chain α/β-type protein that is widely used in biodiesel preparation. Improving the catalytic activity and methanol tolerance of RML is necessary to realise the industrial production of biodiesel.

**Results:**

In this study, a semi-rational design method was used to optimise the catalytic activity and methanol tolerance of ProRML. After N-glycosylation modification of the α-helix of the mature peptide in ProRML, the resulting mutants N218, N93, N115, N260, and N183 increased enzyme activity by 66.81, 13.54, 10.33, 3.69, and 2.39 times than that of WT, respectively. The residual activities of N218 and N260 were 88.78% and 86.08% after incubation in 50% methanol for 2.5 h, respectively. In addition, the biodiesel yield of all mutants was improved when methanol was added once and reacted for 24 h with colza oil as the raw material. N260 and N218 increased the biodiesel yield from 9.49% to 88.75% and 90.46%, respectively.

**Conclusions:**

These results indicate that optimising N-glycosylation modification in the α-helix structure is an effective strategy for improving the performance of ProRML. This study provides an effective approach to improve the design of the enzyme and the properties of lipase mutants, thereby rendering them suitable for industrial biomass conversion.

**Supplementary Information:**

The online version contains supplementary material available at 10.1186/s13068-021-02087-6.

## Background

Biodiesel (fatty acid methyl ester, FAME) is an ideal substitute for fossil fuels as a novel renewable energy source, which comprises low-sulphur, low-carbon, and high-hexadecane contents [1]. Enzymatic biodiesel production has become popular in recent years owing to its mild reaction conditions and low energy consumption [2]. Lipase (EC 3.1.1.3) can hydrolyse the ester bond of the water-insoluble substrate at the substrate–water interface, exhibiting a significant level of activity and stability in a non-aqueous environment; thus, it is widely used in biodiesel production. Compared with the current method of biodiesel production that utilises solid acid and alkali catalysts, the use of lipase reduces a large amount of lipid waste in a sustainable and environmentally friendly manner, provides a potential solution to energy security issues, and offers a new approach to achieve green production of biodiesel [3, 4].

From a practical perspective, the activity, thermostability, and methanol tolerance of lipase are the main reasons that hinder its effective application in the biodiesel field, resulting in prolonged reaction times and increased production costs [5]. Therefore, improving the specific tolerance and catalytic efficiency of the enzyme is key to further expanding the application of lipase. Currently, a semi-rational design approach based on the protein structure is an effective way to improve the properties of enzymes that are based on the modification of several key residues and domains in terms of the structure–function relationship of the enzyme [6–8].

N-Glycosylation is one of the most common and important post-translational modifications of proteins and involves the covalent linkage of carbohydrates on the asparagine residue in the Asn-Xaa-Ser/Thr sequence (where Xaa is any amino acid except Pro) [9]. It is necessary for a variety of functions related to protein secretion, folding, stability, antigenicity, and enzyme activity [10, 11]. The influence of glycosylation modification on enzyme activity is complicated and depends on the glycosylation site, glycoform, and glycan length [12]. Glycosylation at different sites can have opposite effects on enzyme activity. The removal of N-glycan at the N88 position of β-1,4-endoglucanase CTendo45 increased its catalytic activity toward CMC-Na and β-d-glucan; however, the addition of polysaccharides at the N65 position increased its catalytic activity [13]. In most cases, the lack of N-glycosylation reduces enzyme activity, which may be caused by changes in the secondary structure of the protein, as detected by circular dichroism analysis [14]. Conversely, recent studies have reported that the introduction of glycosylation sites increased enzyme activity. The addition of N-glycans in *Rhizopus oryzae* lipase increased the secretion of enzymes, resulting in an increase in catalytic activity from 0.4 U/mL to 207 U/mL [15]. Moreover, N-glycosylation can stabilise proteins expressed in eukaryotic cells because N-glycosylation helps maintain the stability of the natural state of the newly synthesised polypeptide [16]. After introducing two N-glycosylation modifications (Q258N and Q349N) into the *E. coli* appA phytase, the thermostability of appA-Q258N/Q349N was increased by more than 40% compared to that of the wild-type protein, and the melting temperature was increased by 4.5 °C [17]. However, not all glycosylation modifications can improve protein stability, sometimes the presence of glycan even had a negative impact on protein stability [18].

In particular, the influence of N-glycans on enzymes depends not only on the enzyme type, but also on the structural region of the enzyme [19]. Therefore, an effective way to achieve breakthrough results to improve protein secretion, function, and stability more easily is to study the regulation mechanism of glycosylation on protein conformation, reasonably controlling the degree of N-glycosylation and the position of N-glycosylation.

RML is a single-chain α/β-type protein that is widely used in the preparation of structural lipids and biodiesel and the separation of enantiomers of chiral drugs [20, 21]. Liu et al. [22] removed two N-glycosylation sites on the propeptide of RML; the resulting catalytic activity was twice than that of the wild-type ProRML (WT). At present, there are no reports on the effect of the N-glycosylation position on RML with propeptide (ProRML). α-Helix is a common and relatively unchanged secondary structural element in proteins. However, a-spiral bodies are not rigid bodies, and their deformation is important in protein function (for example, a coiled helix) [23]. α-Helix plays an important role in maintaining the tertiary structure, affecting the stability and folding of the protein. Single or multiple mutations may have a significant impact on the overall stability of the protein [24]. In our previous work, two potential N-glycosylation sites on ProRML were removed using site-directed mutagenesis, and the effect of N-glycosylation of the propeptide on ProRML was eliminated. In this study, new glycosylation recognition sequences were constructed in the α-helix structure of its mature peptide through site-directed mutagenesis, and the effects of glycan in this region on the expression, enzyme activity, thermostability, and methanol stability of ProRML were studied. Five N-glycosylation mutants were created in the α-helical structure, which conferred stronger activity and methanol tolerance, thereby providing a promising biocatalyst for a variety of biotechnological applications. Importantly, this work provides a reference for research on N-glycosylation functions of other enzymes and establishes a feasible and effective method for improving the enzyme redesign process.

## Results

### Semi-rational design of N-glycosylation sites in ProRML

In a previous study, we confirmed that ProRML was N-glycosylated and expressed in *P. pastori*s and that N-glycan plays a key role in lipase activity and stability (data not shown). ProRML comprises a propeptide and mature peptide. Propeptides act as intramolecular chaperones to promote the folding of the mature peptide, and the mature peptide performs its biological function after being correctly folded [25]. Therefore, to study the effect of N-glycosylation on ProRML, five N-glycosylation sites were introduced in the α-helix by amino acid substitution: N93 (T93N), N115 (D115N), N183 (L183N), N218 (G218N), and N260 (V260N).

### N-Glycosylation identification of ProRML and its mutants

**Fig. 1:**
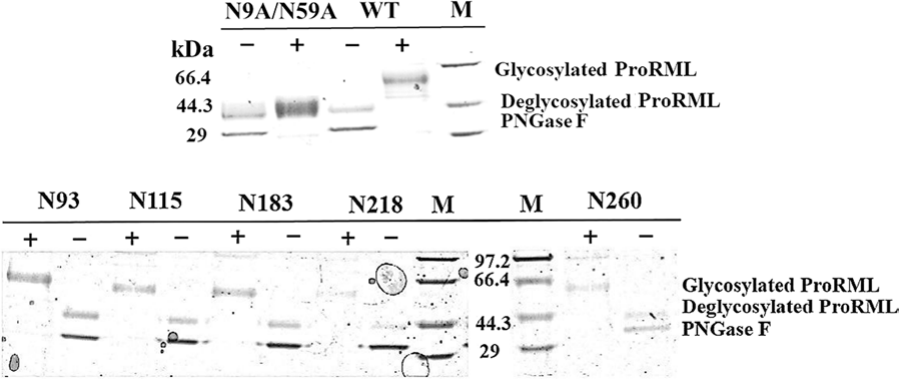
SDS-PAGE analysis of the N9A/N59A and glycosylation mutants. +: processed without PNGase F; −: processed with PNGase F

To test whether the mutant enzymes are N-glycosylated, the purified ProRMLs were incubated with peptide-*N*-glycosidase F (PNGase F). Mobility transfer on sodium dodecyl sulphate-polyacrylamide gel electrophoresis (SDS-PAGE) is one of the simplest methods to assess the degree of deglycosylation [26]. SDS-PAGE showed that the molecular weight of the mutants changed significantly with PNGase F treatment (Fig. 1). Mutants N93, N115, N183, N218, and N260 all showed a band of approximately 55 kDa before PNGase F treatment, while N9A/N59A without glycans, showed a band of approximately 40 kDa. After the mutants were treated with PNGase F, two bands were observed. One was approximately 40 kDa, which is consistent with the molecular weight of N9A/N59A. The other band was approximately 35 kDa, which is consistent with the molecular size of PNGase F. Previous studies have shown that PNGase F can completely remove the N-glycans of glycoproteins [27]. These results indicate that the constructed mutant enzymes were all glycosylated, and the lengths of N-glycans on these mutants were homogeneous.

### Activity and kinetic analysis of glycosylated mutant enzymes

**Fig. 2:**
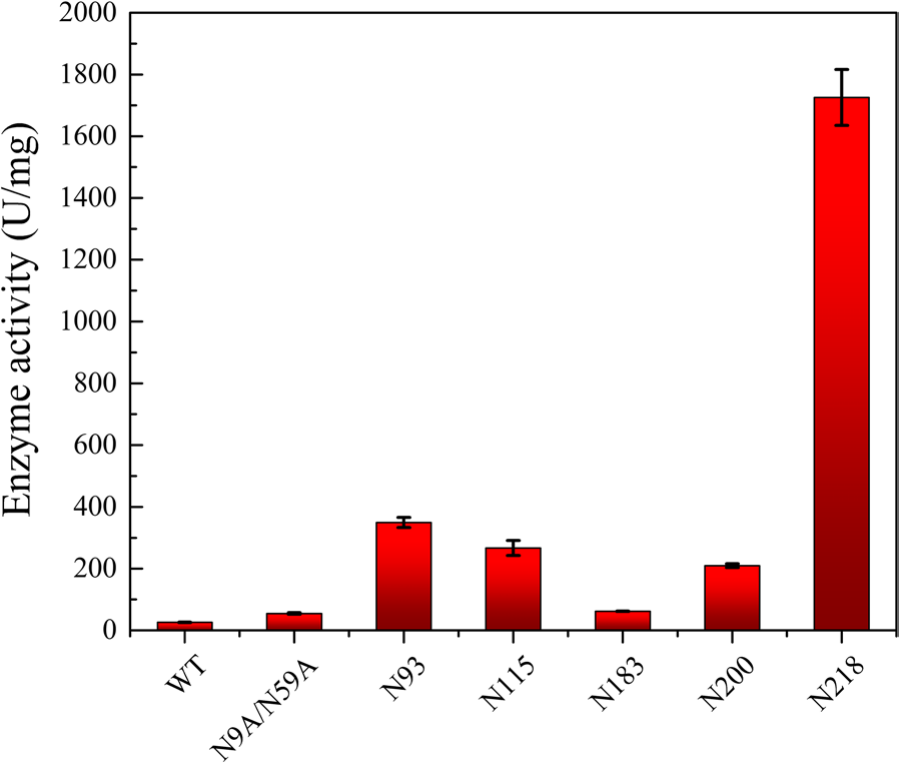
Enzyme activity of the WT, N9A/N59A and glycosylation mutants

Enzyme activity and kinetic constants of WT, N9A/N59A, and N-glycosylation mutants were measured at 40 °C and pH 8.0. As shown in Fig. 2, the enzyme activities of all glycosylation mutant enzymes were higher than those of WT and non-glycosylated N9A/N59A. The maximum enzyme activity of N218 (1725.81 ± 90.56 U/mg) was 66.81 and 31.69 times that of WT and N9A/N59A, respectively. The enzymatic activities of N93, N115, N260, and N183 (349.75 ± 16.36 U/mg, 266.82 ± 24.43 U/mg, 95.36 ± 2.91 U/mg, and 61.62 ± 0.33 U/mg, respectively) were 6.42, 6.42, 4.9, and 1.13 times than that of N9A/N59A, respectively. The Km, kcat, and kcat/Km values of all mutants were greater than those of non-glycosylated N9A/N59A, which showed that the addition of N-glycan to the α-helix structures of the mature peptide reduced the affinity of the enzyme to the substrate, but improved the catalytic efficiency of the enzyme. Mutant N218 has a positive effect on the catalytic activity and catalytic efficiency of ProRML, as its kcat and kcat/Km values were 1049.65 and 26.75 times than those of N9A/N59A, respectively.

### Effect of N-glycosylation on optimal pH and temperature of the lipases

**Fig. 3:**
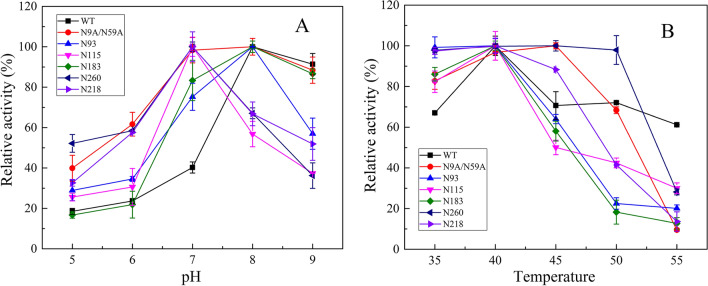
Optimal reaction conditions. The optimal pH (**A**) and temperature (**B**) of the WT, N9A/N59A and its mutants were assessed using para-nitrophenyl palmitate (PNPP) as the substrate

The optimal pH value for enzyme activity was determined over the range of 5.0–9.0. The optimal pH values of N93 and N183 were consistent with those of WT and non-glycosylated N9A/N59A. However, the optimal pH of mutants N115, N218, and N260 decreased from 8 to 7. In addition, most of the mutant enzymes showed more than 80% activity at pH of 7–9 (Fig. 3A). The effect of temperature on enzyme activity is shown in Fig. 3B. The optimum temperature for N260 and N9A/N59A was 45 °C, but the temperature of the WT and other glycosylation mutants was 40 °C. These results indicate that the addition of glycan to the α-helix had little effect on pH and temperature change.

### Thermostability assay of ProRML and its mutants

**Fig. 4:**
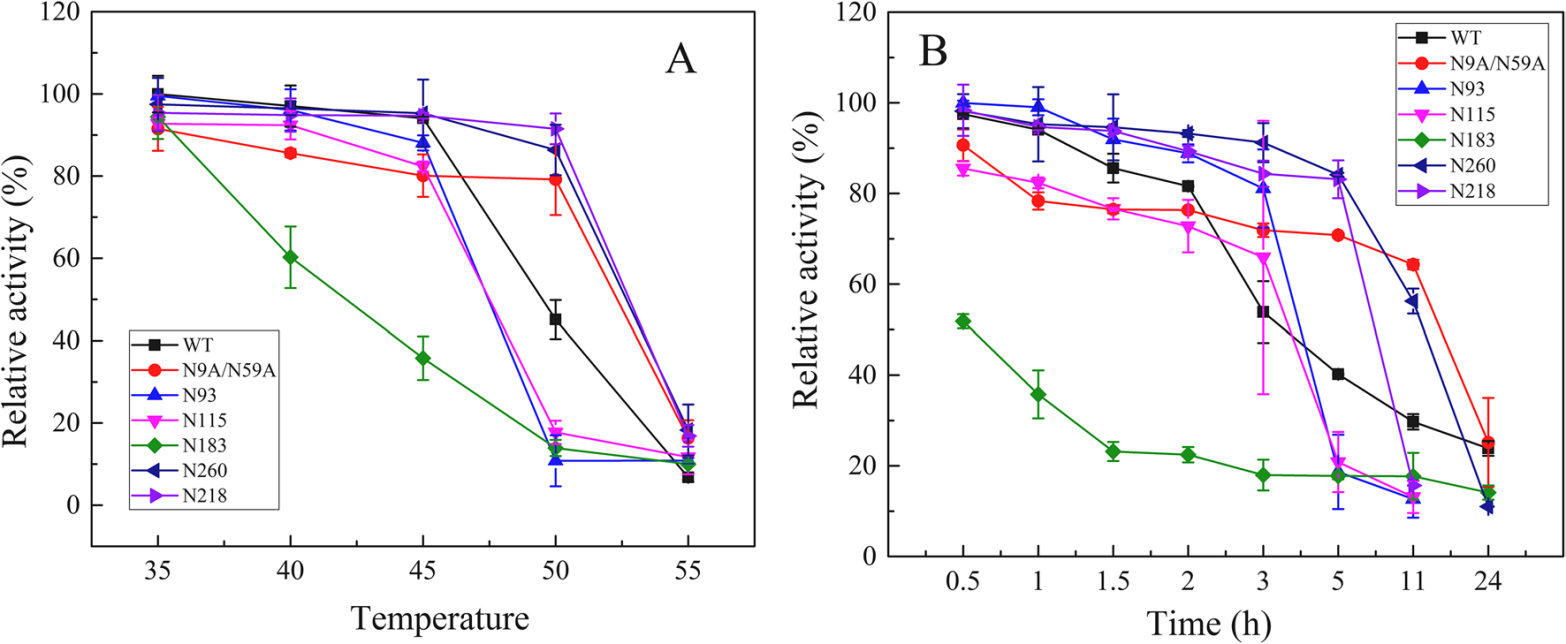
Thermostability assay of ProRML. Thermostability of the enzyme was determined using PNPP as the substrate. The thermostability was measured by evaluating the residual activity after enzyme was pre-incubated for 1 h at different temperatures (**A**) and pre-incubated for 24 h at 45 °C (**B**). The activity of the unheated sample was set to 100%, and the relative hydrolytic activity at each time point is presented as a percentage

To determine the effect of glycan on the thermostability of the enzyme, the residual activities of WT, N9A/N59A, and glycosylation mutants were measured by preincubating at 35–55 °C for 1 h. As shown in Fig. 4A, the residual activities of N260 and N218 incubated at 45 °C for 1 h were 95.27% and 94.71%, respectively, which were higher than those of WT and N9A/N59A. However, the residual activities of N80 and N183 were much lower than those of WT and N9A/N59A. N260 and N218 both maintained more than 85% residual activity after incubation at 50 °C for 1 h, which was higher than that of the WT and N9A/N59A. However, the other mutant enzymes were almost completely inactivated. As the temperature increased, enzyme activity decreased rapidly (Fig. 4A). In addition, the residual enzyme activities of N260 and N218 were greater than 80% after incubation at 45 °C for 5 h, which was higher than the residual activities of WT and N9A/N59A under the same conditions. However, almost all other glycosylation mutant enzymes were inactivated. After incubation at 45 °C for 11 h, all glycosylation mutant enzymes were inactivated, but the non-glycosylated N9A/N59A retained 64% of its residual activity (Fig. 4B). After incubation for 2.5 h at 50 °C, the residual activity of all glycosylation mutant enzymes was less than 30%, although the residual activity of N218 was 29.02%, which was higher than that of WT and non-glycosylated N9A/N59A (Table 1). The results show that the mutants N218 and N260 had better thermostability than WT and the other mutants. Overall, adding N-glycan to the α-helix structure region of mature peptides had no effect on the temperature stability of ProRML and showed no consistent results, which may be related to the position of the N-glycan on the surface of the lipase and the distribution of nearby amino acids [28].

**Table 1:** Kinetic parameters and thermostability at 50 °C of WT, N9A/N59A, and glycosylation mutants

Mutants	Km (mM)	kcat (min^−1^)	kcat/Km (min^−1^ mM^−1^)	Thermostability at 50 °C (2.5 h)
WT	1.16	105.29 ± 2.96	90.49 ± 2.55	26.92 ± 2.26
N9A/N59A	0.35	50.61 ± 2.31	145.35 ± 6.65	25.77 ± 9.83
N93	4.70	3816.33 ± 157.15	812.33 ± 33.45	12.79 ± 6.36
N115	0.64	435.51 ± 16.61	679.43 ± 25.91	8.62 ± 4.31
N183	0.36	71.15 ± 0.38	195.49 ± 1.05	14.66 ± 0.85
N260	1.25	293.27 ± 8.94	235.56 ± 7.18	10.47 ± 4.31
N218	13.2	51,322.99 ± 1563.66	3888.11 ± 118.46	29.02 ± 5.52

### Methanol tolerance assay of ProRML and its mutants

**Fig. 5:**
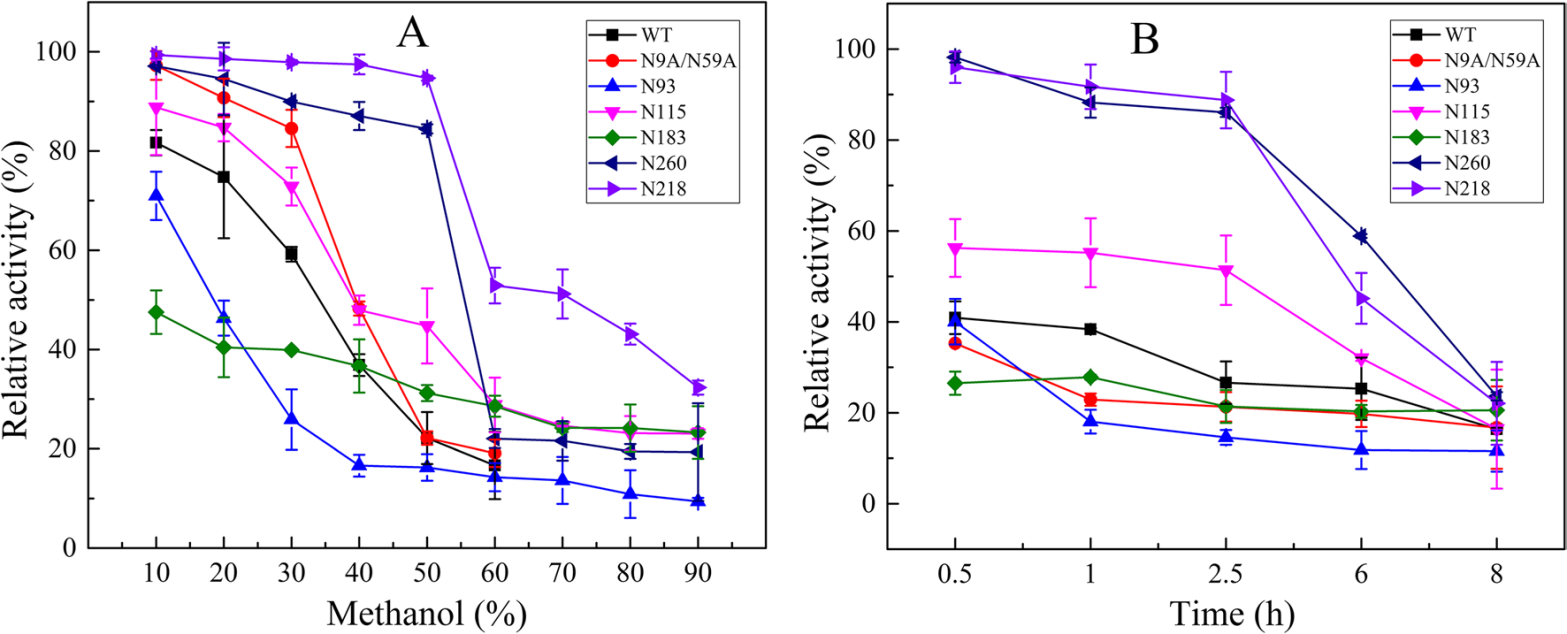
Methanol tolerance of ProRML. Methanol tolerance of the enzyme was determined using PNPP as the substrate. The methanol tolerance was measured by evaluating the residual activity after enzyme was pre-incubated for 1 h at different methanol concentrations (**A**) and pre-incubated for 8 h at a 50% methanol concentration (**B**). The activity of the unheated sample was set to 100%, and the relative hydrolytic activity at each time point is presented as a percentage

*Rhizomucor miehei* lipase is widely used for preparing biodiesel with good methanol tolerance. Therefore, improvement in its methanol tolerance would make it even more desirable within the biodiesel industry. The methanol tolerance was investigated at 10–90% methanol concentration, and the results are shown in Fig. 5A. After incubating in 50% methanol for 1 h, the residual activities of N260, N218, N115, and N183 were 84.45%, 94.67%, 44.77%, and 31.21%, respectively, which were all higher than those of WT (16.66%) and N9A/N59A (22.15%). However, the residual activity of N93 was slightly lower than that of N9A/N59A. The residual activity of N218 in 60% and 80% methanol solutions were 52.90% and 43.11%, respectively. After incubating for 2.5 h in 50% methanol solution, the residual enzyme activities of N218, N260, and N115 were 88.78%, 86.08, and 51.36%, respectively, which were all much higher than the residual activities of N9A/N59A. However, the other glycosylation mutants were lower than the N9A/N59A. When incubation was continued for 6 h more, the residual activities of N260, N218, and N115 were 58.88%, 45.16%, and 32.00%, respectively, which were higher than those of N9A/N59A. After incubation for 8 h, however, the residual activities of all mutant enzymes were less than 25%. These data show that mutants N260 and N218 have the best thermostability and methanol tolerance out of the tested mutants, and were noticeably improved from WT.

### Biotransformation of colza oil and waste soybean oil to produce FAME

**Fig. 6:**
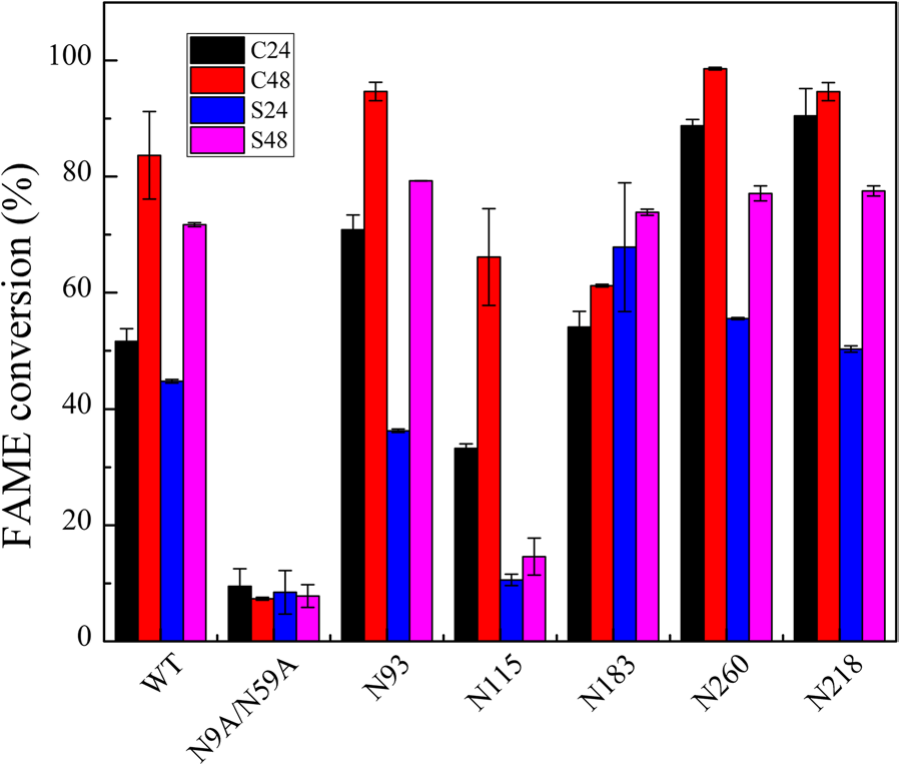
Biotransformation of FAME. Biotransformation of 5 g colza oil and waste soybean oil to FAME when 0.2 g methanol (molar ratio of 6:1 methanol to oil) is added in one-shot using 5 mL WT and mutant enzymes. C24: biotransformation of colza oil for 24 h; C48: biotransformation of colza oil for 48 h; S24: biotransformation of waste soybean oil for 24 h; S48: biotransformation of waste soybean oil for 48 h

To evaluate the ability of N-glycosylated mutants to produce biodiesel, the enzymatic hydrolysis of colza oil and waste soybean oil was analysed. Biotransformation of FAME was carried out with methanol in one step [methanol-to-oil ratio of 6:1 (g/g)] and liquid enzyme (5 mL) at 35 °C for 24 h (Fig. 6). The biodiesel yields of all glycosylation mutant enzymes were much higher than those of non-glycosylated N9A/N59A using colza oil or waste soybean oil for 24 h. The conversion rates of N260 and N218 increased from 9.49% to 88.75% and 90.46%, respectively, with colza oil as the raw material. In addition, the FAME conversion rates of N93, N260, and N218 were 94.67%, 98.59%, and 94.62%, respectively, for 48 h, and it is notable that these mutants converted almost all of the substrate. The FAME conversion rates of N183, N260, and N218 with waste soybean oil increased from 8.45% to 67.84%, 55.54%, and 50.28%, respectively, after 24 h. After 48 h, the biodiesel conversion rates of the other glycosylation mutants increased to more than 70%, except for N115. Compared with colza oil, the biodiesel conversion time of waste soybean oil took longer and the conversion rate was lower. This may be because there are more acidic substances in the waste soybean oil, which reduces the transesterification reaction ability of the glycosylation mutant enzymes.

## Discussion

Lipase is an important biocatalyst that converts oil into green and pollution-free biodiesel [2]. Therefore, improving the catalytic activity, methanol tolerance, and transesterification efficiency of lipase has attracted widespread attention [29]. At present, structure-based semi-rational design is an effective strategy for improving enzyme performance, which is undertaken by adding modifications to particular positions along the enzyme [30, 31].

Glycosylation is one of the most common modifications of heterologous protein expression in *P. pastoris* [32]. Owing to the important application of N-glycan in biotechnology, its influence on enzyme structure and activity has been widely reported [33]. The carbohydrate portion of the protein has many functions, one of which is to stabilise the protein conformation. In this study, a semi-rational design strategy was adopted to improve the biochemical performance of ProRML by introducing N-glycosylation into the α-helix structure of a mature peptide. Through glycosidase PNGase F digestion and SDS-PAGE analysis, the mutants N93, N183, N115, N218, and N260 were all glycosylated (Fig. 1).

The addition of large glycans attached to the protein backbone will change the structure of the protein, which in turn changes its function. All mutants possessed higher enzyme activity than WT and N9A/N59A, and the addition of N-glycan at the N218 site significantly increased the catalytic activity of ProRML (Fig. 2). This may be because the addition of glycan causes the substrate to stay longer in the catalytic pocket of the enzyme, and the enzyme has a stronger ability to bind to the substrate. Therefore, N-glycans located at specific positions of proteins may be essential for control of folding kinetics and secretory pathways of the protein [34, 35]. In addition, factors such as the number, location and the surface morphology of glycosylation sites, and the distance from the active site to glycosylated site can affect the number of hydrogen bonds and hydrophobic interactions between glycans and proteins, which in turn change the protein structure [10, 36]. In the hydrophobic microenvironment of the active site, the presence of hydrophilic glycans may be conducive to strengthen the polar groups interactions and facilitate catalysis. Furthermore, mutants N218, N93, and N115, whose polar amino acids were mutated to polar asparagine, exhibited higher activities than those of N260 and N183, where hydrophobic amino acids were mutated to asparagine.

In addition, our data indicated that glycosylation modification had a slight effect on the optimal pH and temperature of ProRML. Previous studies have shown that N-glycosylation significantly affects enzyme activity [15]. The N-glycosylation sites of exo-inulinase from *Kluyveromyces cicerisporus* were mutated, and the enzyme activity of the obtained mutant was lost [37]. Marcelo et al. [38] redesigned N-glycosylation sites in a GH3 β-xylosidase, which improved enzymatic efficiency and revealed that N-glycans increase the overall flexibility of the protein, especially the structural elements near the catalytic pocket, thereby enhancing the substrate binding performance. The addition of N-glycan to the N260 and N218 positions resulted in an increase in thermostability; however, the addition at other positions resulted in a significant decrease in thermostability. This indicates that the glycan position has an appreciable impact on the thermostability of ProRML. Previous research has shown that the excellent stability introduced by additional glycosylation is closely related to entropy, which is largely dependent on the location of the glycosylation site [39]. In addition, the surface polarity of the glycosylated protein would change significantly, the tertiary structure would be extended, and some hydrophobic amino acids would be exposed to a more hydrophilic environment. The higher stability of N260 and N218 may be related to their surrounding amino acid positions and properties. Conjugated glycans may have a strong clamping effect and tighten the spatial conformation of the enzyme by increasing the configuration entropy [39].

Similar to the thermostability, the methanol tolerance of N260 and N218 was significantly improved by the addition of N-glycan. N260 and N218 retained 58.88% and 45.16% of the residual activity incubation in 50% methanol for 6 h, whereas WT, N9A/N59A, and the other mutants were almost completely inactivated. Compared with thermostability, N-glycan added to mature peptides is more conducive to improving the methanol tolerance of RML. Conjugated N-glycans are considered strong clamps, which tighten the spatial conformation of the enzyme by increasing the configuration entropy [39]. In addition, through CD analysis, Owens et al. [40] confirmed that hydrogen bonds were formed between oligosaccharides and peptides, and the oligosaccharide residues formed a hydrophilic shielding layer in the helical structure, which may account for the increased conformational stability of glycosylated mutants.

Lipases with high catalytic activity, thermostability, and methanol tolerance are the best biocatalysts for the preparation of biodiesel. By adding methanol once every 24 h, the FAME yields of most glycosylation mutants exceeded 60%, with some being as high as 90%. Compared with the traditional method of adding methanol in batches, the one-time addition of methanol simplifies the preparation process and reduces the reaction time of biodiesel.

## Conclusion

From a practical point of view, promising enzyme candidates should exhibit extraordinary catalytic activity, thermostability, and methanol tolerance. In this study, we first explored the effect of N-glycosylation on ProRML in the α-helix structure of mature peptides and obtained five mutants with improved catalytic activity, thermostability, methanol tolerance, and biodiesel conversion rate. The modification of N-glycans in ProRML had a greater impact on methanol stability than on thermostability. These results indicate that optimising the N-glycosylation modification in the α-helix structure is an effective strategy for improving the performance of ProRML. This study holds considerable significance for improving the biochemical performance of enzymes through rational design and engineering of homologous and structurally similar enzymes.

## Materials and methods

### Materials

*Pichia pastoris* GS115 (*P. pastoris*) (Invitrogen, USA) was used as an exogenous expression host for recombinant protein production. The Fast Mutagenesis System Kit (TransGen, China) was used to introduce N-glycosylation sites using primers for each site. The primers were synthesised by Sangon Biotech (China) and are listed in Additional file 1: Table S1. All chemicals were of analytical grade.

### Mutagenesis of ProRML

By analysing the tertiary structure of the mature peptide of the wild-type ProRML (WT, GenBank: A02536), candidate mutation sites T93, D115, L183, G218, and V260 were determined in combination with the N-glycosylation signature sequence N-X-S/T (X is any amino acid except Pro). Using the plasmid pPIC9K/ProRML as a template, the N9A/N59A mutation was obtained by removing two glycosylation sites through site-directed mutagenesis. The primers used are listed in Additional file 1: Table S1. Using the pPIC9K/N9A/N59A plasmid as a template, the expression plasmids of mutants N93(T93N), N115(D115N), N183(L183N), N218(G218N), and N260(V260N) were obtained by site-directed mutagenesis, and the mutants were transformed into *E. coli* DMT. Positive transformants were selected in LB medium supplemented with 50 μg/mL kanamycin, and gene sequencing was performed using the 3′-AOX primers and α-factor (Additional file 1: Table S1).

### Transformation and heterologous expression in *P. pastoris*

The recombinant expression plasmids were linearised using the restriction enzyme SacI and transformed into *P. pastoris*. The transformants were seeded on minimal glucose (MD) and then screened on tributyrin plates. Finally, according to the *P. pastoris* expression manual, the mutants were cultured in BMGY and agitated for 44 h (200 rpm, 30 °C); subsequently, they were switched to BMMY and agitated for 120 h. After every 24 h, 0.5% methanol was added.

### Purification and SDS-PAGE analysis

The cell-free supernatant was collected via centrifugation (8000 rpm, 10 min, 4 °C) and then concentrated using a 10-kDa ultrafiltration membrane (4000 rpm, 30 min, 4 °C). The crude enzyme was purified using a HisTrap-HP column (GE Healthcare, UK). The binding buffer (20 mM PBS buffer, 500 mM NaCl, 20–40 mM imidazole, pH 8.0) was used to wash away impurities at a flow rate of 3 mL/min. The target proteins were washed with a linear gradient of elution buffer (20 mM PBS buffer, 500 mM NaCl, 500 mM imidazole, pH 8.0) at a flow rate of 1 mL/min. Protein concentration was determined using a BCA protein assay kit (Sangon Biotech, China). SDS-PAGE was performed to separate proteins based on molecular mass, and then the proteins were observed using Coomassie blue R-250 (Sigma-Aldrich, MO, USA) staining [41, 42].

### Glycoproteins digestions with PNGase F

Protein samples were mixed in 10× glycoprotein denaturation buffer and distilled and deionised water and boiled for 10 min. The denatured glycoproteins were then treated with PNGase F at 37 °C for 1 h in 10× G7 reaction buffer containing 10% NP40. The glycosylation of the proteins was detected using SDS-PAGE.

### Enzyme activity assays

The para-nitrophenyl palmitate method was used to detect enzyme activity. The reaction mixture contained 20 μL of 20 mM PNPP and 20 μL of diluted enzyme solution in 50 mM PBS buffer (pH 8.0). After incubating at 40 °C for 5 min, 500 μL of 0.5 M trichloroacetic acid was added to stop the reaction. Then, 400 μL reaction solution was mixed with 140 μL of 0.5 M NaOH, and then the absorbance of the reaction mixture was measured at a wavelength of 410 nm. One unit (U) was defined as the amount of enzyme that released 1 μmol of PNP per minute. All experiments were performed in triplicates.

### Kinetic characterisation

The reaction was performed in 50 mM PBS buffer (pH 8.0) at 40 °C for 5 min using PNPP at various concentrations (0–400 μmol). The kinetic parameters were calculated using the Michaelis–Menten equation [43].

### Biochemical characterisation

The optimal pH value of enzyme activity was determined in 50 mM PBS buffer (pH 5.0–9.0) at 40 °C. The optimum temperature within the range of 35–55 °C was determined at pH 8.0.

Thermostability was determined by measuring the residual activity after the enzyme was pre-incubated at different temperatures (35–55 °C for 1 h, 45 °C for 24 h, and 50 °C for 8 h). The methanol tolerance was observed by measuring the residual activity after preincubation at methanol concentrations of 10–90% and 60% for 1 and 8 h, respectively. Thermostability and methanol tolerance were evaluated based on the ratio of the residual activity to the initial activity. The relative activity is shown as a percentage of the released PNP production, with a maximum value of 100%.

### Biotransformation of grease into biodiesel

Five mL of crude enzyme was added to 5 g of colza oil or waste soybean oil, while 0.2 g of methanol (methanol: oil molar ratio of 6:1) was also added to the mixtures. The reaction was performed at 35 °C at 200 rpm for 48 h, and samples were taken at intervals of 24 h. FAME was determined using gas chromatography (GC).

The conversion rates of FAME were determined via GC analysis, which was performed using a Shimadzu GC-2010 Plus GC system (Japan) equipped with a flame ionisation detector and a capillary column (DB-WAX, 30 m × 0.25 μm × 0.25 μm). The reaction sample was diluted in 5 mL *n*-hexane (containing 0.2 mg *n*-hexadecane) as an internal standard. The analysed FAMEs included methyl myristate (C14:0), methyl palmitate (C16:0), methyl stearate (C18:0), methyl oleate (C18:1), methyl linoleate (C18:2), methyl linolenate (C18:3), methyl eicosanoate (C20:0), methyl eicosenoate (C20:1), and methyl behenate (C22:0). Methyl heptadecanoate (C17:0) was used as the internal standard. The output of the FAME was then calculated according to Eq. 1 [44]:

1 $${\text{Yield}} = \frac{{\sum {\text{Ci}} \times V}}{m} \times 100\% ,$$

where *m* is the mass of the FAMEs, Ci is the concentration of the FAMEs in the sample, and *V* is the volume of the sample.

## Supplementary Information

**Additional file 1: Table S1. **The primers list.

## Data Availability

All data generated or analysed during this study are included in this published article and its Additional file.

## Abbreviations

FAME: Fatty acid methyl ester
GC: Gas chromatography
PNGase F: Peptide-*N*-glycosidase F
PNPP: para-Nitrophenyl palmitate
RML: *Rhizomucor miehei* lipase
ProRML: *Rhizomucor miehei* lipase with propeptide
WT: Wild-type ProRML
SDS-PAGE: Sodium dodecyl sulphate-polyacrylamide gel electrophoresis
