# Supplementary material for: Improved methanol tolerance of Rhizomucor miehei lipase based on N‑glycosylation within the α-helix region and its application in biodiesel production
Source: Biotechnol Biofuels. 2021 Dec 15;14:237. doi: 10.1186/s13068-021-02087-6 (PMC8675521; doi:10.1186/s13068-021-02087-6)
Supplement: Supplementary file 1 — Additional file 1: Table S1. The primers list. [file 13068_2021_2087_MOESM1_ESM.docx]

Table S1 The primers lists

| Mutations | Mutagenic primers |
| --- | --- |
| N9-F | 5‘-AAGAGACAGTCCNNKTCCACCGTTGACTCTTTGCC-3‘ |
| N9-R | 5‘-GAMNNGGACTGTCTCTTGATTGGAACATGGTG-3‘ |
| N59-F | 5‘-GGCTCTGNNKGCTACTTCCTACCCAGATTCCGT-3‘ |
| N59-R | 5‘-AAGTAGCMNNCAGAGCCATACCGTACTTGGTTT-3‘ |
| N93-F | 5‘-CTACACCaacTTGTCCGCTAACTCCTATTGCA-3‘ |
| N93-R | 5‘-CGGACAAgttGGTGTAGTAGGTCAACTCGTTGATCT-3‘ |
| N115-F | 5‘-TCCATTGCaacGCTACCGAAGACTTGAAGATCATCA-3‘ |
| N115-R | 5‘-TCCATTGCaacGCTACCGAAGACTTGAAGATCATCA-3‘ |
| N183-F | 5‘-GGGTTTCaacGATTCCTACGGAGAAGTGCAGAA-3‘ |
| N183-R | 5‘-GGGTTTCaacGATTCCTACGGAGAAGTGCAGAA-3‘ |
| N218-F | 5‘-TTTGGGAaacGCTACAGCTTTGTTGTGCGCTT-3‘ |
| N218-R | 5‘-TTTGGGAaacGCTACAGCTTTGTTGTGCGCTT-3‘ |
| N260-F | 5‘-CTACGTTaacTCCACCGGTATCCCATACAGAA-3‘ |
| N260-F | 5‘-CTACGTTaacTCCACCGGTATCCCATACAGAA-3‘ |
